# The Direct Effects of Air Pollutant Exposure from Industrial Complexes on Chronic Respiratory Diseases in Local Residents: A Population-Based Cohort Study

**DOI:** 10.3390/ijerph22050666

**Published:** 2025-04-23

**Authors:** Ho-Hyun Kim

**Affiliations:** Department of Nano-Chemical, Biological and Environmental Engineering, SeoKyeong University, 139, Seogyeong-ro, Seongbuk-gu, Seoul 02717, Republic of Korea; ho04sh@skuniv.ac.kr

**Keywords:** air pollution, emission sources, factory, epidemiological correlation, village

## Abstract

New evidence suggests that industrial areas may have a negative impact on the chronic respiratory disease burden among residents who live more than 10 km away compared to residents who live within at least 5 km. The main cause of air pollution in factories was found to be benzene, and its regional relevance was confirmed. This is the result of many studies confirming that benzene is a substance that directly affects the respiratory system, and we aim to maintain continuous observation in the future. Using South Korean health insurance data, we retrospectively followed a cohort of residents living near a factory and monitored them from 2002 to 2022. The aim of this study was to identify respiratory symptoms, such as cough, sore throat, nasal discomfort, and shortness of breath, as the most common signs of air pollution. The results of the measurements around the factory location (within 1 km) showed a high epidemiological correlation with Hwagok-ri and the village hall due to the high benzene concentration (average high concentration (11 cases) of 0.82 ± 0.46 ppb) at the factory, as well as around the business location (within 1 km) in Dokgot-ri and the village hall (average high concentration (8 cases) of 32.29 ± 19.73). The conclusion is that respiratory diseases are linked to the severity of air pollution in the areas surrounding this industrial complex.

## 1. Introduction

There have been many studies on long-term exposure to heavy metals generated in workplaces; however, this is the first in which a follow-up observation was conducted on hospital treatment for respiratory diseases caused by long-term exposure. This methodology is essential for utilizing data to inform regulations and policies for the management of industrial complexes [1]. The purpose of this study is to address the fact that the risk of exposure to benzene is not well established. This study also suggests that benzene exposure in a single workplace may be associated with respiratory diseases other than asthma and rhinitis [2].

To preserve national competitiveness, South Korea has built industrial complexes in Yeocheon, Gwangyang, and Ulsan since the 1970s, operating under the tenets of economic development and guaranteeing a foundation for industrialization [3,4,5,6]. Although the development of these industrial areas has been essential for the country’s economic expansion, the surrounding neighborhoods experience air and soil pollution, unpleasant odors, and heavy metal emissions [7,8,9]. The potential harm that environmental pollution can cause to local populations is worrisome [10,11,12,13].

The results of detailed examinations of the sources of pollutants within the primary categories of emission sources, manufacturing combustion, manufacturing processes, energy transportation and storage, waste treatment, agriculture, and other pollutants, confirmed the classification technique regarding emission sources [14,15,16,17]. The various categories of emission classifications are as follows: the production of asphalt concrete; mining; food and beverage manufacturing; textile manufacturing; the manufacturing of fur and suede clothing and products; the manufacturing of leather, bags, shoes, pulp, and paper products; publishing; compounds and chemicals; rubber and plastic; the manufacturing of non-metallic mineral products; the manufacturing of assembled metal products; medical optical equipment; and the manufacturing of household waste [18,19,20,21,22,23]. This classification approach was derived from the results of research on commercial, residential, and industrial wastewater treatments to validate a preliminary investigation [24,25].

Gyeongsangbuk-do’s CO (carbon monoxide), VOCs (Volatile Organic Compounds), and NH3 (ammonia) levels have been verified to be higher than those in the metropolitan area and are typically higher compared to the entire region because of the distribution of sources of emissions [26,27].

There are very large amounts of different types of volatile organic compounds. Both large and relatively low quantities of these compounds are created in common locations, such as automobiles, petrol stations, laundromats, and photo development businesses, as well as in facilities, such as in the paint and petrochemical industries [28,29,30].

Nonetheless, because volatile organic compounds are found in both discharges of pollutants into the atmosphere and outdoor environments, they contribute to photochemical reactions on the Earth’s surface, exacerbating global warming, destroying the ozone layer, and posing health risks due to their toxicity and carcinogenicity; as such, interest in them is steadily increasing [31]. Techniques for the partial quantification and qualitative identification of individual compounds have developed rapidly because of the need to gather and analyze trace amounts of organic substances that are prevalent in the environment [32]. Therefore, the environmental monitoring of these compounds is extremely important. Respiratory allergies are becoming more prevalent in South Korea because of changes in the levels of air allergens and air pollution [33,34]. This has significant ramifications for future management and research, in addition to social and financial costs.

Symptoms related to the respiratory system, including coughing, throat and nose discomfort, and breathing difficulties, are the most common signs of air pollution. The symptoms of patients with chronic lung disease or asthma may worsen [35,36]. Epidemiological studies indicate an increase in hospitalizations for heart and respiratory conditions, as well as a higher risk of death in patients with lung and heart conditions [37,38]. According to research, air pollutants are the reason for the increase in lung cancer cases; however, since carcinogens such as benzene and PAHs (polycyclic aromatic hydrocarbons) are present in the air, it is difficult to completely rule out the idea that air pollutants are connected to the development of cancer [39,40].

According to the World Health Organization, environmental factors are responsible for 25–33% of diseases in industrialized countries [41,42]. Children and vulnerable populations are disproportionately affected. Furthermore, a greater understanding of the importance of environmental preservation over economic growth has resulted in the environment being prioritized over economic progress [43]. The need for a receptor-centered indoor environment policy to improve public health has arisen from the shortcomings of current media-centered environmental management, which is unable to meet public health expectations and adapt to the emergence of new environment-related diseases [44,45]. Since most people nowadays spend over 90% of their time indoors, the significance of home and indoor air quality is paramount [46]. As such, it is crucial to control dangerous air pollutants in at-risk areas [47]. Pollutants in the air can affect exposure and health. The harmful effects of air pollution on human health must be considered in the context of the broad category of environmental pollutants [48,49]. The main purpose of this study is to address the lack of clear management of benzene, which affects health, in the national management target area over the past ten years. Therefore, this study is expected to provide basic data for identifying environmental measurements and health indicators in specific areas through follow-up observations of local residents, obtaining the consent of residents who have lived in the area for up to twenty years.

## 2. Method

### 2.1. Study Area

**Figure 1:**
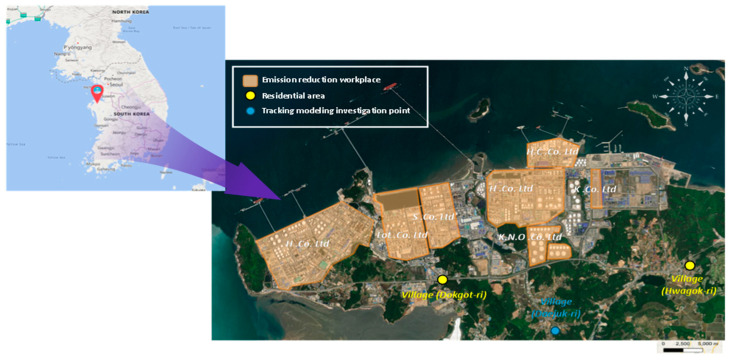
Location of the study area (emissions, residential areas, traceability modeling).

Figure 1 shows the characteristics of areas with health impacts due to exposure to major chemicals from a single petrochemical plant in South Korea. This project involved conducting measurement and analyses with the cooperation of 10 industrial plants, as shown in Figure 1. In Daejuk-ri, Daesan-eup, Seosan-si, and Chungcheongnam-do, industrial complexes are set to develop over an area of 9,120,000 m^2^. Hyundai Oilbank, LG Chemical, Lotte Chemical, Hanwha Total, KCC, South Korean National Oil Corporation, and over 60 small and medium-sized businesses are located in Daesan, one of the country’s three main petrochemical complexes. Development management is required, because more industrial plants will be added in the future, endangering the health of the community. Previous health examinations in the Daesan Industrial Complex region revealed a high incidence of cancer and asthma; respiratory symptoms, such as cough, dyspnea, wheezing, and asthma were significantly prevalent. Ongoing follow-up observations are required to establish a causal link according to the features of the environment in this area.

### 2.2. Air Pollution Monitoring

Using ten sizable workplaces as measuring points, the processes used in handling, storage, and shipping facilities were examined. Ten workplaces completed the survey with the cooperation of the Ministry of Environment. In order to avoid interfering with the business operations of the companies, the survey was conducted only during certain dates and times designated for facilities handling benzene (chemicals such as benzene, toluene, and xylene are often commonly detected VOCs). The status of the emission sources, including marine and land facilities, was determined. These sources included manufacturing, transportation, and shipping operations, which release volatile organic compounds during the process of oil refining. The survey was conducted in the vicinity of the complexes, with particular attention to roadside and workplace fences. In addition, measurements and analyses were conducted in the village halls of Dokgot-ri, Hwagok-ri, and Daejuk-ri (residential areas). To identify hazardous volatile organic chemicals in ambient air, samples were collected in accordance with the Air Pollution Process Testing Standard (ES 01804.2a) and subjected to solid adsorption [50]. Approximately 100 or more samples were collected from ten separate workplaces, and approximately 40 samples were taken from the nearby village.

### 2.3. Sample Analysis Method

#### 2.3.1. Sample Collection

Volatile organic compounds (VOCs, SIBATA Sigma, MP-Pump, MP-∑30KNII) were collected using a sorbent tube (Tenax-TA) with the pump suction method (adsorption tube method) at a collection flow rate of 100 mL/min for a collection time of 0.5 hr. In line with the Air Pollution Process Testing Standard (ES 01804.2a), the solid adsorption method was used for testing for the presence of hazardous volatile organic compounds (VOCs) in the ambient air by collecting samples. The sampling procedure involved checking the suction pump flow rate, installing an anemometer and suction pump, connecting the adsorption tube, and collecting samples at 0.1 to 0.5 L per minute for 10 min. The wind direction, wind speed, and temperature were recorded, and the samples were stored in a refrigerated state and then transported to an analytical institution for analysis. The adsorption tube was installed on the pump, and samples were collected from a height of 1.2 m. The wind direction and wind speed were measured while the samples were collected. The above procedure was conducted taking into account the conditions of the affected residents and the geographical characteristics of the area, which exhibits a high prevalence of chronic diseases. Samples were collected during the operating hours of the various business premises. Additionally, the collection process was conducted with consideration of the general environmental characteristics, such as the short duration, the extreme occurrence of chemicals in the surrounding area, and the exclusion of many business processes.

#### 2.3.2. Sample Analysis

Eighteen volatile organic compounds were purchased from Sigma-Aldrich (St. Louis, MO, USA), Kemidas (Gunpo, Gyeonggi, Republic of Korea), Burdick & Jackson (Muskegon, MI, USA), J. T. Baker (Center Valley, PA, USA), and Junsei Chemical (Chuo-ku, Tokyo, Japan) and were used as standard materials. A standard stock solution (10,000 mg/L in dimethylformamide) corresponding to 250 mg of each analyte was prepared in a 25 mL volumetric flask; dimethylformamide was filled up to the mark, a stopper was installed, and the flask was shaken for mixing. The sample pretreatment method involved the transfer of all the adsorbents in the adsorption tube to a 2 mL vial, followed by the quick addition of 0.5 mL of carbon disulfide and 25 μL of mixed internal standard solution (1.0 mg/L in dimethylformamide). Thereafter, the the stopper was closed and the sample was left at room temperature for 30 min before injecting into the gas chromatograph (GC) inlet and analyzing using a gas chromatograph mass spectrometer (mass spectrometer) and a gas chromatograph tandem mass spectrometer. The GC-MS conditions corresponded to the experimental conditions for 15 VOCs, except for vinyl chloride and 1,3-butadiene. For the simultaneous analysis of volatile organic compounds, a GC (7890A Series GC, Agilent Technologies, Palo Alto, CA, USA) was used. An MS of 5975C was used for the measurements. The conditions for the GC-MS instrumental analysis are listed in Table 1 [51].

### 2.4. Establishment of a Standardization Technique for Monitoring Emission Concentrations

**Figure 2:**
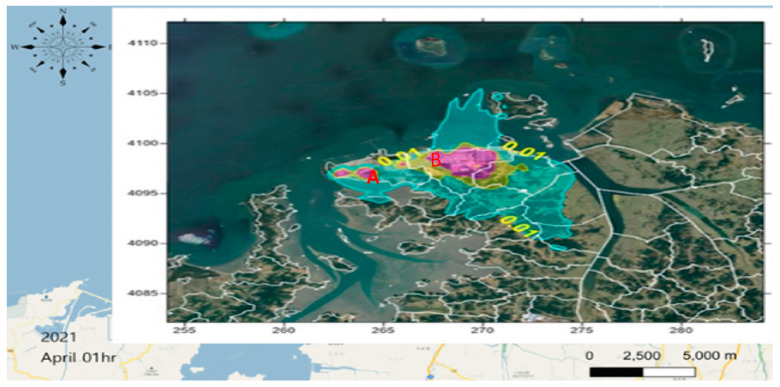
Daesan area annual average atmospheric diffusion modeling concentration field (benzene, unit: μg/m^3^).

Daesan-eup and Seosan-si are national industrial complexes in South Korea that are surrounded by sea and require environmental pollution management. At ten important industrial plants within these industrial complexes, measurement sites were selected, and the characteristics of residential areas and schools within a 5 km radius from the emission sources were confirmed. Benzene is produced from production facilities, storage tanks, and wastewater treatment facilities; hence, it is difficult to predict its provenance. In order to identify the pollutant, monitoring was conducted 24 h a day for 2 weeks per season. We factored in meteorological conditions, i.e., hourly wind direction and speed data, to monitor benzene levels along the fence line of one workplace and utilized an atmospheric diffusion model to evaluate the characteristics of pollutants flowing from that workplace into a home. Information on the major wind characteristic indicators, such as the overall 3-year and seasonal prevalent winds and average wind speed, is presented in Figure 2. In Figure 2, the closer the color is to red, the greater the risk of exposure, centered on a 0.01 μg/m^3^ concentration of benzene. The results were verified according to causality by considering the outdoor measurement results (benzene) and meteorological input data (temperature, wind direction, wind speed, and humidity measured by AWS). Figure 2 presents the annual average air dispersion model for the Daesan region in Korea. The figure highlights areas with benzene concentrations reaching 0.01 μg/m^3^, marked in red. Notably, elevated concentrations are observed near major industrial complexes, Hyundai Oilbank (A) and Lotte Chemical (B), indicating the severity of emission characteristics associated with storage and handling processes at these facilities.

Northwesterly winds occur throughout the year in the Daesan area (near the industrial complex), accounting for 17.4% of the total wind direction frequency. The northwesterly wind reflects the influence of the sea breeze and can be interpreted as a wind field characteristic combined with the seasonal wind. In the case of wind speed, 29.2% of the winds appeared in the 0.5–2 m/s range, showing the maximum frequency; the average wind speed was found to be 3.02 m/s. The frequency of stagnant air (<1 m/s), which is an important wind indicator related to dispersion, was 0.5%. In other words, 29.8% of the area near Daesan had weak wind speeds of less than 2 m/s.

### 2.5. Statistical Analysis

The South Korean National Health Insurance Service (NHIS) database from 2002 to December 2022 was used. NHIS provides the SAS Enterprise Guide. NHIS is a single payer system for healthcare services managed by the government, covering approximately 97% of the South Korean population. The SAS Enterprise Guide (SAS Institute Inc., Cary, NC, USA) is an integrated software suite used for advanced analytics, business intelligence, data management, and predictive analytics using statistical methods. The SAS software (SAS Enterprise Guide 7.1) can be used through a graphical interface and the SAS programming language or Base SAS. The Health Insurance Corporation analyzed the data using this program (SAS Enterprise Guide). The global database includes an eligibility database (age, sex, socioeconomic variables, type of eligibility, residential area code, etc.), a medical treatment database (date of visits, primary and secondary diagnosis codes, etc., submitted by medical service providers), a health examination database (results of general health examinations and questionnaires), and a medical institution database (type of institutions, number of physicians, etc.). Those who had resided in the area for more than five years were considered eligible. January 1st of the following year after the confirmation of the five-year residence was set as the baseline date of observation, and those who had lived in both the exposure and the control area were excluded from entering the cohort [52]. Based on a cohort investigation of the occurrence of chronic diseases, continuous observation was possible in the survey and control areas. The period of residence was analyzed based on accumulated data. Among these, a retrospective cohort study was conducted on respiratory diseases. The target areas were analyzed by grouping them into exposure and control areas. In order to calculate the applied results, a Cox proportional hazard model was used as a statistical analysis method, and the hazard ratio of living in the survey area compared to living in the control area was calculated using data for 23 chronic diseases. The analysis was conducted using a Chi-square test considering the characteristics of the exposure area and control area. A statistical method was applied to the data of the corporation, where the observation points were available, and a customized database was used, making it possible to model the risk of chronic disease and cancer. Adjusted models, such as the crude model, age, sex, and income, were analyzed, and cases with low incidences of chronic diseases were excluded from the analysis. Finally, regarding the period of residence of the participants, the analysis considered those who had lived in the area for less than 10 years as dropouts.

### 2.6. Moving Window Positive Matrix Factorization

Window Positive Matrix Factorization (PMF) is a method of moving existing PMF input data by specific cycle units, configuring multiple datasets, and performing PMF modeling for each input dataset [53,54,55,56]. In this study, measurements and analyses were conducted every 2 weeks from 18 November 2022 to February 2023, and the data were moved once a month. When the PMF model was applied using the input data organized in this manner, multiple contributions of pollutants corresponding to a specific day were derived, providing the advantage of being able to calculate the standard deviation of the model results. In addition, because modeling was performed by moving each date, it was possible to identify additional pollutants on a specific day. The PMF model may be expressed as the following determinant.

X = GF + E

- X: n × m matrix (n: number of samples, m: number of chemical species analyzed).
- G: n × p matrix (n: number of samples, p: number of pollutants) which represents the emission amount for a specific pollutant source, i.e., the contribution of the pollutant source to the receptor.
- F: p × m matrix (p: number of pollutants, m: number of chemical species analyzed) which contains information on the emission source and is often called a pollutant source classification table (Source Profile).
- E: the residual matrix, which can be expressed as the following equation.

$$E_{j}=X_{\mathrm{ij}}-\sum_{k=1}^{p}G_{\mathrm{ih}}F_{\mathrm{hi}}\;(i=1\;\sim \;n,\;j=1\;\sim \;m,\;k=1\;\sim \;p)$$

The PMF model was designed so that the G (contribution) value and the F (pollutant source classification table) value would have positive values and the model solution could be obtained under the condition of minimizing Q(E) regarding the G and F values.

$$Q=\overset{n}{\underset{i=1}{\sum }}\overset{n}{\underset{i=1}{\sum }}{\left(\begin{array}{c} E_{\mathrm{ij}}\\ U_{\mathrm{ij}} \end{array}\right)}^{2}$$

Here, μ_ij_ represents the uncertainty of X_ij_.

The modeling temperature, wind direction, wind speed, humidity, dew point temperature, cloud cover, cloud height, and local pressure were measured at regular weather stations within the area, and temperature, wind direction, wind speed, and humidity were measured at AWS. The high-altitude weather data comprised temperature, wind direction, wind speed, dew point temperature, and pressure data, measured twice a day at the nearest high-altitude weather station. The observation data information and location information used in the industrial complex are as follows (Table 2 and Table 3).

## 3. Results

### 3.1. Measurement Results Using the Air Diffusion Model in the Industrial Plant and Surrounding Areas

**Figure 3:**
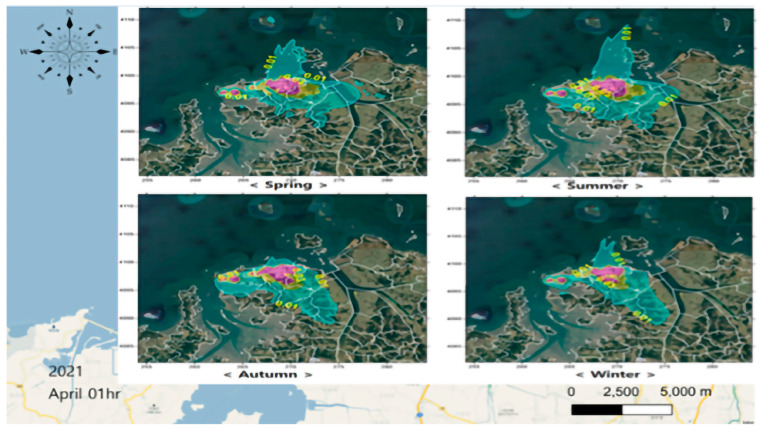
Daesan area annual average atmospheric diffusion modeling for benzene concentration field (unit: ppb).

The total amount of air emissions according to the Pollutant Release and Transfer Registers, managed by the South Korean government, was applied to the Clean Air Policy Support System data (Air Pollutants Emission Inventory, emission input data of the modeling prediction method) based on the distribution of pollutants and the characteristics of the emission sources. The results of estimating benzene emissions in the Daesan area are presented. The spatial distributions of the benzene point and surface sources were confirmed. In the case of benzene point sources, the emission points were confirmed to be concentrated to the east of the business site, while the grid surface sources were evenly distributed within the Daesan area. In spring and summer, the main wind direction was southwesterly, with considerable easterly and southerly winds. Thus, the pollutants diffused toward the southwest and west, and, unlike in other seasons, also spread northward. In autumn, the concentration spread to the southeast and west owing to the influence of northwesterly and easterly winds. In winter, the northwesterly winds were stronger compared with other seasons, and the pollutants were found to spread to the southeast (Figure 3). The Daesang Industrial Complex is a unique case in South Korea; in contrast to other business complexes, it is a single business site for petroleum manufacturing. It could serve as a good example of the creation of a management system based on the health statuses of local residents.

### 3.2. Characteristics of the Area Surrounding the Plant 

**Figure 4:**
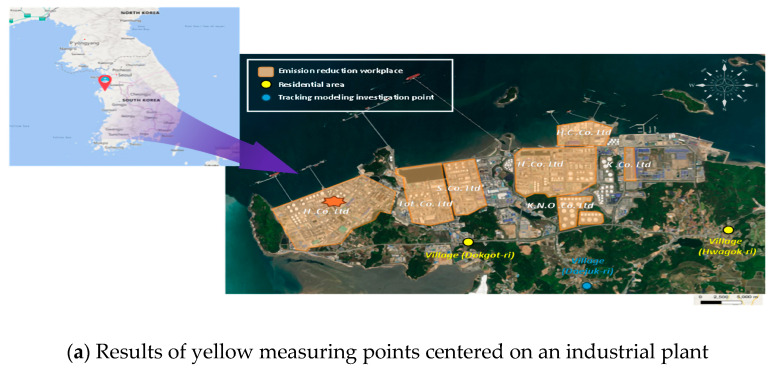

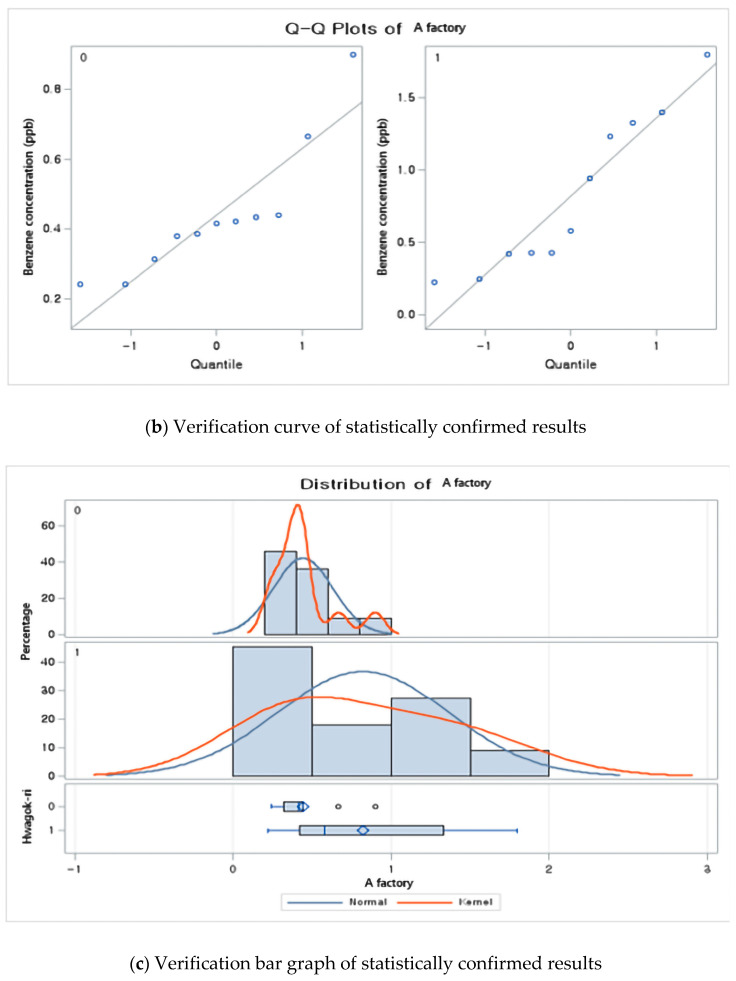
Statistical analysis of measurement results of the area surrounding the industrial plant and Hwagok-ri.

We checked the national measurement network data on benzene, which is directly related to negative health effects of residents due to exposure to air pollution in the workplace and surrounding areas; see Figure 3. As shown in Figure 2, elevated benzene concentrations represented in red were observed around major industrial facilities such as Hyundai Oilbank (A) and Lotte Chemical (B), confirming the severity of emission characteristics associated with storage and handling processes. Prevailing northwesterly winds during spring and winter and southeasterly winds during summer and autumn were identified as influencing factors that contribute to seasonal variations in the dispersion of these emissions. Accordingly, we checked the results of the relationship between the concentrations of pollutants in the industrial plant and the distance from the source, focusing on the results that clearly showed a link between the presence of benzene and respiratory diseases. Statistically significant results were obtained by comparing the results of the measurement of the area surrounding Plant A (red left point) with epidemiological data for Hwagok-ri and the village hall (Average concentrations of benzene (11 cases) 0.82 ± 0.46 ppb, Average Low (11 cases) 0.44 ± 0.31 ppb; *p* > 0.0403; Figure 4 and Table 4).

As illustrated in Figure 4, the emission characteristics of Hyundai Oilbank were identified with high benzene concentrations under northwesterly and northeasterly winds at wind speeds of 1–3 m/sec. These findings indicate that the elevated concentrations are a result of storage and handling activities within the facility.

**Figure 5:**
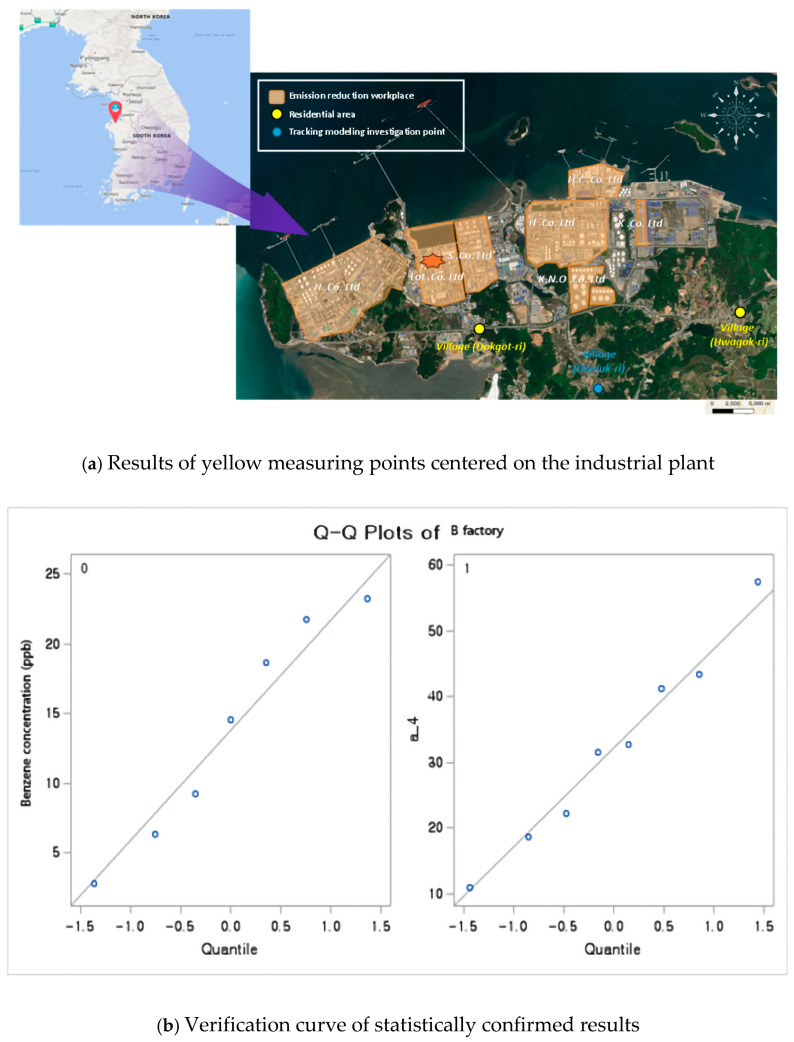

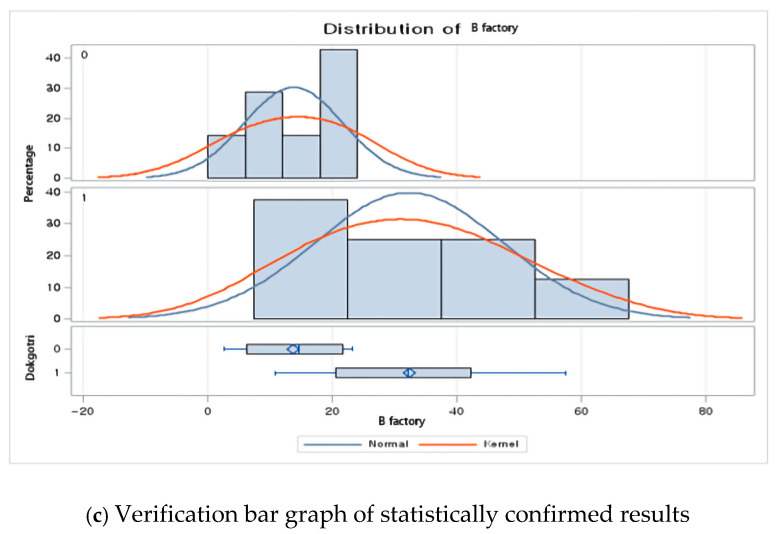
Statistical analysis of measurement results of the area surrounding the industrial plant and Dokgotri.

We confirmed that Workplace B, an industrial complex on the right side of Figure 3, was an area with a high concentration of industrial waste which could result in significant damage to residents. When compared to the measurement results around Workplace B in Dokgot-ri and the village hall (Average High (8 cases) 32.29 ± 19.73, Average Low (7 cases) 13.79 ± 6.47), a statistically significant result was confirmed (*p* > 0.0115; Figure 5 and Table 5). As shown in Figure 5, the emission characteristics of Lotte Chemical revealed elevated benzene concentrations under westerly wind conditions at wind speeds ranging from 0 to 2 m/sec. This pattern is attributed to storage and handling activities within the facility.

### 3.3. Results of a Cohort Analysis of Health Insurance Data from Industrial Complexes (Dokgotri and Hwagok-ri)

The total number of participants in the Daesang Industrial Complex was 33,070, including 21,636 in the control area (>10 km from the factory; 54.88% men and 45.12% women), 7222 in the medium-exposure area (within 5 km from the factory; 53.78% men and 56.51% women), and 4212 in the high-exposure area (within 2 km from the factory; 46.22% men and 43.49% women). The average age was 30.00 ± 19.85 years in the control area, which was significantly higher than 29.22 ± 20.10 years in the medium exposure area and 32.13 ± 21.07 years in the high exposure area (*p* > 0.01826). In terms of age distribution in the control area, the 20–39 years age group was the largest (37.68%), followed by those under 20 years (32.07%), 40–64 years (23.78%), and ≥65 years (6.48%). In the medium-exposure area, <20 years of age group was the largest (35.67%), followed by 20–39 years (33.48%), 40–64 years (24.73%), and ≥ 65 years (6.12%). In the high-exposure area, the 20–39 years age group was the largest (1512; 35.90%), followed by the < 20 years group (28.87%), the 40–64 years group at 25.97, and the ≥ 65 years group (9.26%). According to the contrast and high exposure, the age distribution was significantly higher in the 20–39 years age group (*p* > 0.00001). Household income was highest in the 3rd and 2nd quartiles in the contrast area (28.53%) and lowest in the 4th (24.16%) and 1st (21.39%) quartiles. The middle exposure area was also confirmed to be the highest in the 4th quartile (56.61%), followed by the 3rd (17.83%), 2nd (13.94%), and 1st (11.62) quartiles. In the high-exposure area the highest income was observed in the 3rd quartile (27.84%), followed by the 4th (26.98%), 2nd (24.92%), and 1st quartile (20.26%), showing a significantly higher distribution of high-income quartiles in the high-exposure area (*p* > 0.00001; Table 6).

Subjects in the exposed areas were identified as having general health problems The results from the follow up of local residents among the 23 participants who had chronic diseases are shown in Table 6. Among participants with chronic diseases, those who had lived in industrial complex areas for more than 5 years had respiratory diseases. The incidence of acute upper respiratory infections (J00–J06) was significantly higher in the medium-exposure area (84.16%) than in the control area (82.71% (*p* > 0.0001). This analysis was conducted based on a diagnosis made at a hospital visit. Other diseases of the upper respiratory tract (J30–J39) were significantly higher in the high-exposure area (37.20%) than in the control area (36.46% (*p* > 0.0001). The incidence characteristics of other acute lower respiratory infections (J20–J22), chronic lower respiratory diseases (J40–J47), and asthma (J45) were confirmed to be the opposite, i.e., between the exposure and control areas (Table 7).

According to the results of other respiratory diseases, the incidence of chronic rhinitis (J31) was 69.39% in the control area, 71.51% in the intermediate-exposure area, and 69.83% in the high-exposure area, and was significantly higher in the exposed area. The incidence of respiratory system disease (J00–J99) was 93.74% in the control area and 94.91% in the intermediate-exposure area, which was significantly higher (*p* > 0.00798). The incidence of respiratory diseases (J00–J99) was 93.74% in the control area and 94.91% in the intermediate-exposure area, which was significantly higher (*p* > 0.00028). On the other hand, in R05, it was confirmed that the Control area was statistically insignificant compared to the Exposed area (Table 8).

### 3.4. Results of Workplace Emissions Correlation and Contribution Priorities

By analyzing the results of the concentrations of volatile organic chemical pollutants in the villages and workplaces, the contribution of exposure was determined to confirm the correlation between the causality of respiratory diseases in the factory and village. This confirmed the contribution of benzene, among other volatile organic chemicals; it was confirmed that benzene had the second highest impact overall. Moreover, it is suggested that the presence of benzene will be a factor in the deterioration of health due to long-term exposure in the future.

**Figure 6:**
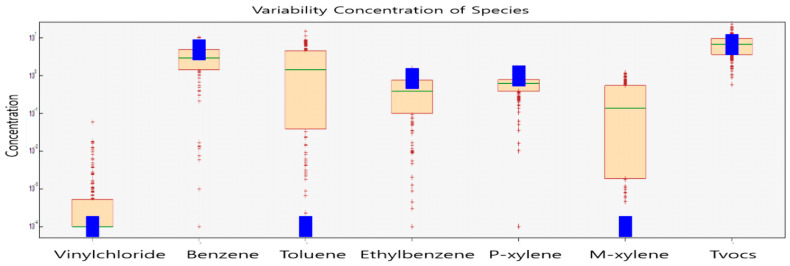
Variability results depending on concentrations of different pollutants at the industrial plant.

It was found in results that considered only the characteristics of benzene, excluding other individual variables that, in addition to exposure to low concentrations, long-term exposure to high concentrations can cause respiratory diseases. The results of the measurements of volatile organic chemicals according to the correlations between villages and factories were used to identify the contribution of benzene by source, considering the variability in the ratios and concentrations of chemicals in the workplace. The results of the main variables regarding concentration and ratio were the same for a factory located within 1 km of the industrial plant as those of a factory located at a distance greater than 1 km; the contribution rate was confirmed in the order of tvocs (50.0%) > benzene (36.4%) > *p*-xylene (7.4%) > ethylbenzene (6.3%); additional compounds were confirmed to be vinyl chloride, toluene, and m-xylene at 0% (Figure 6, Table 9).

## 4. Discussion

Daesang and Seosan-si are special national industrial complexes in South Korea that are surrounded by sea. At both sites, environmental pollution management is necessary. Using ten sizable workplaces as measuring points, the states of handling, storage, and shipping facilities were examined. Workplaces included manufacturing, transportation, and shipping operations, which release volatile organic compounds via the processing of oil refineries. Among the emissions these industrial complexes, benzene is produced from production facilities, storage tanks, and wastewater treatment facilities, so it is difficult to predict and measure its provenance. In order to identify the pollutants, monitoring was conducted for 24 h a day for 2 weeks per season. Northwesterly winds are mainly prevalent throughout the year in the Daesan area (near the industrial complex). Based on a cohort investigation of the occurrence of chronic diseases, continuous observation was possible in the survey and control areas. The period of residence was analyzed based on the accumulated data. A retrospective cohort study was conducted on respiratory diseases. In order to calculate the results, a Cox proportional hazard model was used as a statistical analysis method, and the hazard ratio of living in the survey area compared to that of living in the control area was calculated using cohorts with 23 chronic diseases. The spatial distributions of benzene point and surface sources were assessed. This confirmed that the concentration of benzene components gradually decreased with distance via diffusion. In spring and summer, the main wind direction was southwesterly, with considerable easterly and southerly winds. Similarly, the diffusion of pollutants proceeded in the southwest and west, and, unlike in other seasons, it spread northward as well. In autumn, pollutants spread to the southeast and west, which are the windward sides. In winter, compared with other seasons, northwest winds prevailed at a high speed, and the pollutants were found to spread to the southeast. Accordingly, we checked the results to evaluate the relationship between distance from the industrial complex and concentration in inhabited areas, focusing on the results that clearly showed a connection between the presence of benzene and the occurrence of respiratory diseases [57,58]. When comparing the results of the measurements from the areas surrounding Workplace A, we observed a high epidemiological correlation between Hwagok-ri and the village hall in terms of the concentrations of benzene (Average High (11 cases) 0.82 ± 0.46 ppb, average low (11 cases) 0.44 ± 0.31 ppb). We observed statistically significant results regarding exposure concentrations. In the measurement results from the areas surrounding Workplace B in Dokgot-ri and the village hall (Average High (8 cases) 32.29 ± 19.73, average low (7 cases) 13.79 ± 6.47), a statistically significant result was confirmed. This confirmed that the same components were consistently being emitted. The total number of participants in the Daesang industrial complex was 33,070, comprising 21,636 in the control area (>10 km from the factory), 7222 in the medium-exposure area (within 5 km from the factory), and 4212 in the high-exposure area (within 2 km from the factory). In terms of sex, 54.88% were men and 45.12% women in the control area, 53.78% were men and 56.51% women in the medium-exposure area, and 46.22% were men and 43.49% women in the high-exposure area. The average age was 30.00 ± 19.85 years in the control area, which was significantly higher than 29.22 ± 20.10 years in the medium exposure area and 32.13 ± 21.07 years in the high exposure area. Household income was highest in the 3rd and 2nd quartiles in the main area (28.53%), and the lowest in the 4th (24.16%) and 1st quartiles (21.39%). The high-exposure area was the highest in the 3rd quartile (27.84%), followed by the 4th quartile (26.98%), 2nd quartile (24.92%), and 1st quartile (20.26%), indicating a significantly higher distribution of high-income quartiles in the high-exposure area. In the case of respiratory diseases among the participants who had lived in industrial complex areas for more than 5 years, the incidence of acute upper respiratory infections (J00–J06) was significantly higher in the medium-exposure area (84.16%) than in the control area (82.71%). Other diseases of the upper respiratory tract (J30–J39) were significantly more common in the high-exposure area (37.20%) than in the control area (36.46%). The incidence of chronic rhinitis (J31) was 69.39% in the control area, 71.51% in the intermediate-exposure area, and 69.83% in the high-exposure area, and was significantly higher in the exposed area. The incidence of respiratory system disease (J00–J99) was 93.74% in the control area and 94.91% in the intermediate-exposure area, which was significantly higher. The results at the factory located within 1 km of the industrial plant were the same as those for the factory location more than 1 km away; the contribution rate was confirmed to be: tvocs (50.0%) > benzene (36.4%) > *p*-xylene (7.4%) > ethylbenzene (6.3%), and vinyl chloride, toluene, and m-xylene (0%). This is the result of a comprehensive survey conducted with the consent of all local residents. It was confirmed that distance from the workplace influence the characteristics of chronic diseases caused by exposure to benzene.

The emission reduction plan for the development of South Korea’s national industrial complexes is used in guidelines as a causal health indicator. In South Korea, it is used as a good indicator for tracking emission sources and the causality of health problems. 

This study presents the following observations. First, considering that there are emissions of VOCS but that information on acceptable emission concentrations (in workplaces and surrounding areas) is insufficient in South Korea, there are limitations in establishing remedial systems. Second, there are no regulations for monitoring emission reduction effects; such regulations will become necessary in the future. Finally, the Ministry of the Environment should publish data so that various reviews can be prepared; as a consequence, the results of monitoring reports and emission reduction efforts submitted by workplaces could be reviewed and improved. The main limitation of the present research was a lack of cooperation due to conflicts between local residents and businesses,. Additionally, the health data had limitations regarding security, leading us to make various assumptions in terms of statistical explanations. In this regard, we will strive to conduct thorough research by relaying public opinions to the government in the future. Considering that in South Korea it is possible to survey 100% of the target population in a given region but it is not possible to include any correction variables, it is believed that government cooperation with future research will contribute to the quality of the obtained data.

## 5. Conclusions

The spatial distributions of the benzene point and surface sources were confirmed. In the spring and summer, the main wind direction was southwesterly, with considerable easternly and southernly winds. It was confirmed that areas affected by the wind had higher incidences of chronic diseases. In the results for the area surrounding Workplace A (Hwagok-ri and the village hall), high concentrations of benzene (Average High (11 cases) 0.82 ± 0.46 ppb, Average Low (11 cases) 0.44 ± 0.31 ppb) were observed. Similarly, in the area surrounding Workplace B (Dokgot-ri and the village hall) high concentrations of benzene (Average High (8 cases) 32.29 ± 19.73, Average Low (7 cases) 13.79 ± 6.47) were confirmed. In the case of respiratory diseases among the participants who had lived in industrial areas for more than 5 years, the incidence of acute upper respiratory infections (J00–J06) was significantly higher in the medium-exposure area (84.16%) than in the control area (82.71%). Other diseases of the upper respiratory tract (J30–J39) were significantly more prevalent in the high-exposure area (37.20%) than in the control area (36.46% (*p* < 0.0001). The incidence of other acute lower respiratory infections (J20–J22), chronic lower respiratory diseases (J40–J47), and asthma (J45) were confirmed to show the opposite trend between the exposure and control areas. The results from a factory located within 1 km of the industrial plant were the same as those of a factory located more than 1 km away. The contribution rate was as follows: tvocs (50.0%) > benzene (36.4%) > *p*-xylene (7.4%) > ethylbenzene (6.3%), and vinyl chloride, toluene, and M-xylene (0%). This is the result of a comprehensive survey conducted with the consent of all participants. It is believed that the prevalence of chronic diseases caused by benzene exposure may be affected by distance from the aforementioned industrial areas.

## Figures and Tables

**Table 1:** Gas chromatography experimental conditions for volatile organic compound analysis.

Parameter	Condition				
**Column**	HP-INNOWAX (60 m × 0.25 mm I.D × 0.25 μm, filmthickness)				
**Carrier Gas flow**	He at 0.9 mL/min.				
**Injection mode**	Split (5:1)				
**Injection volume**	2 μL				
**Injection port temp**	250 °C				
***Trans*ferline temp**	260 °C				
**Oven temp. program**	initial temp (°C)	initial time (min)	rate (°C/min)	temp (°C)	time (min)
	35	0	5	190	0
			30	250	3

**Table 2:** Period to which WRF and CALMET/CALPUFF modeling was applied.

Modeling Period	WRF Modeling	1 January 2021 (UTC+09)–1 January 2021 (UTC+09)
	CALMET/CALPUFF Modeling	1 January 2021 (UTC+09)–1 January 2021 (UTC+09)

**Table 3:** Meteorological modeling input conditions.

Contents (Item)		Details
		Modeling Area (30 km × 30 km)
Grid origin (Reference Point)	UTM-X (km)	254.123
	UTM-Y (km)	4082.131
Grid spacing (ΔX) (m)		300
Domain Size	Nx (No. of x grid Cells)	100
	Ny (No. of y grid Cells)	100
Projection		Universal Transverse Mercator (UTM)
UTM zone		52 (Northern)
No. of Vertical layers		8
Cell face heights (m)		0, 20, 50, 100, 200, 300, 500, 1000, 3000
Bias		−1, −1, −1, −0.8, 0, 0.5, 1, 1
Time zone		UTC+0900

**Table 4:** Average (upper and lower) results of the area surrounding the plant and Hwagok-ri.

Hwagok-ri	N	Business Site A (Red Left)	
		Mean ± S.D.	Pr > |t|
Average High	11	0.82 ± 0.46	0.0403
Average Low	11	0.44 ± 0.31	

**Table 5:** Average (upper and lower) results of the area surrounding the plant and Dokgotri.

Dokgotri	N	Business Site B (Red Right)	
		Mean ± S.D.	Pr > |t|
Average High	8	32.29 ± 19.73	0.0115
Average Low	7	13.79 ± 6.47	

**Table 6:** General characteristics of all participants in two industrial complexes (Dokgotri and Hwagok-ri), low-, high-, and medium-exposure areas (Unit, No. [%]).

		Control Area	Exposed Area		* *p*-Value
		Low	Medium	High	
**Total (people)**		21,636	7222	4212	
**Gender**	**Man**	11,874 (54.88)	3884 (53.78)	2380 (56.51)	0.01826
	**Female**	9762 (45.12)	3338 (46.22)	1832 (43.49)	
**Age (years)**	**Average**	30.00 ± 19.85	29.22 ± 20.10	32.13 ± 21.07	0.00001
	**under 20 years old**	6938 (32.07)	2576 (35.67)	1216 (28.87)	0.00001
	**20–39 years old**	8152 (37.68)	2418 (33.48)	1512 (35.90)	
	**40–64 years old**	5144 (23.78)	1786 (24.73)	1094 (25.97)	
	**65 years or older**	1402 (6.48)	442 (6.12)	390 (9.26)	
** **Income quartile, n (%)**	**1st quartile**	4548 (21.39)	830 (11.62)	844 (20.26)	0.00001
	**2nd quartile**	5268 (28.53)	996 (13.94)	1038 (24.92)	
	**3rd quartile**	5918 (28.53)	1274 (17.83)	1160 (27.84)	
	**quartile**	5012 (24.16)	4044 (56.61)	1124 (26.98)	

* Chi-square test; ** Income decile: Health insurance premium 20 deciles divided into quartiles.

**Table 7:** Descriptive summary results for respiratory diseases between control and exposure areas of industrial complexes (Unit, No. [%]).

		Control Area	Exposed Area		* *p*-Value
		Low ^(3)^	Medium ^(2)^	High ^(1)^	
**Acute upper respiratory infections (J00–J06)**		13,796	4318	2842	
**Number of subjects (persons, %)**	**No**	2386 (17.29)	684 (15.84)	564 (19.85)	*p* > 0.0001
**Result of occurrence**	**Yes**	11,410 (82.71)	3634 (84.16)	564 (19.85)	
**Other diseases of upper respiratory tract (J30–J39)**		20,488	6734	4032	
**Number of subjects (persons, %)**	**No**	13,018 (63.54)	4132 (61.36)	2532 (62.80)	0.0056
**Result of occurrence**	**Yes**	7470 (36.46)	2602 (38.64)	1500 (37.20)	
**Other acute lower respiratory infections (J20–J22)**		15,280	4628	3132	
**Number of subjects (persons, %)**	**No**	3140 (20.55)	980 (21.18)	704 (22.48)	0.0489
**Result of occurrence**	**Yes**	12,140 (79.45)	3648 (78.82)	2428 (77.52)	
**Chronic lower respiratory diseases (J40–J47)**		20,860	6900	4062	
**Number of subjects (persons, %)**	**No**	12,624 (60.52)	4350 (63.04)	2618 (64.45)	*p* > 0.0001
**Result of occurrence**	**Yes**	8236 (39.48)	2550 (36.96)	1444 (35.55)	
**Asthma (J45)**		20,772	6894	4036	
**Number of subjects (persons, %)**	**No**	16,930 (81.50)	5660 (82.10)	3292 (81.57)	0.5363
**Result of occurrence**	**Yes**	3842 (18.50)	1234 (17.90)	744 (18.43)	

* Chi-square test; exposure area: Daesang Industrial Complex; (1) High-exposure area (within 2 km from the factory); (2) medium-exposure area (within 5 km from the factory); and (3) Control area (>10 km from the factory).

**Table 8:** Descriptive summary between control and exposed areas in two industrial complexes (Unit, No. [%]) for other respiratory diseases.

		Control Area	Exposed Area		* *p*-Value
		Low ^(3)^	Medium ^(2)^	High ^(1)^	
**Chronic rhinitis (J31)**		18,602	5974	3666	
**Number of subjects (persons, %)**	**No**	5694 (30.61)	1702 (28.49)	1106 (30.17)	0.00798
**Result of occurrence**	**Yes**	12,908 (69.39)	4272 (71.51)	2560 (69.83)	
**Diseases of the respiratory system (J00–J99)**		10,290	3024	2138	
**Number of subjects (persons, %)**	**No**	644 (6.26)	154 (5.09)	168 (7.86)	0.00028
**Result of occurrence**	**Yes**	9646 (93.74)	2870 (94.91)	1970 (92.14)	
**Cough (R05)**		21,538	7194	4196	
**Number of subjects (persons, %)**	**No**	20,126 (93.44)	6740 (93.69)	3954 (94.23)	0.1518
**Result of occurrence**	**Yes**	1412 (6.56)	454 (6.31)	242 (5.77)	

* Chi-square test; exposure area: Daesang Industrial Complex; (1) high-exposure area (within 2 km from the factory); (2) medium-exposure area (within 5 km from the factory); and (3) control area (>10 km from the factory).

**Table 9:** Contribution rate results according to the emission source and measured concentration in the workplace.

Chemical	Factor1	Contribution Ratio	Factor2	Contribution Ratio	Factor3	Contribution Ratio	Factor4	Contribution Ratio	Factor5	Contribution Ratio	Factor6	Contribution Ratio
**Vinylchloride**	0.000	0.0	0.000	0.0	0.000	0.0	0.000	0.0	0.377	2.7	0.003	0.0
**Benzene**	4.843	36.4	3.568	9.2	0.762	23.1	1.254	20.9	2.656	19.2	0.000	0.0
**Toluene**	0.000	0.0	14.918	38.5	0.000	0.0	0.000	0.0	2.566	18.5	10.482	47.0
**Ethylbenzene**	0.837	6.3	0.427	1.1	0.878	26.6	0.067	1.1	0.883	6.4	0.013	0.1
**P-xylene**	0.980	7.4	0.000	0.0	0.000	0.0	0.196	3.3	0.005	0.0	0.363	1.6
**M-xylene**	0.000	0.0	0.434	1.1	0.000	0.0	1.487	24.7	0.430	3.1	0.272	1.2
**Tvocs**	6.658	50.0	19.374	50.0	1.657	50.3	3.004	50.0	6.922	50.0	11.147	50.0

## Data Availability

The dataset analyzed during the current study are available from the corresponding author on reasonable request.
